# DNA restriction fragment length polymorphism analysis of *Mycobacterium tuberculosis *isolates from HIV-seropositive and HIV-seronegative patients in Kampala, Uganda

**DOI:** 10.1186/1471-2334-9-12

**Published:** 2009-02-05

**Authors:** Benon B Asiimwe, Moses L Joloba, Solomon Ghebremichael, Tuija Koivula, David P Kateete, Fred A Katabazi, Alexander Pennhag, Ramona Petersson, Gunilla Kallenius

**Affiliations:** 1 Department of Microbiology, Tumor and Cell Biology, Karolinska Institutet, SE-171 77, Stockholm, Sweden; 2 Department of Medical Microbiology, Makerere University Medical School, P O Box 7072, Kampala, Uganda; 3 Department of Bacteriology, Swedish Institute for Infectious Diseases Control, SE-171 82, Solna, Sweden; 4 Centre for Microbiological Preparedness, Swedish Institute for Infectious Diseases Control, SE-171 82, Solna, Sweden

## Abstract

**Background:**

The identification and differentiation of strains of *Mycobacterium tuberculosis *by DNA fingerprinting has provided a better understanding of the epidemiology and tracing the transmission of tuberculosis. We set out to determine if there was a relationship between the risk of belonging to a group of tuberculosis patients with identical mycobacterial DNA fingerprint patterns and the HIV sero-status of the individuals in a high TB incidence peri-urban setting of Kampala, Uganda.

**Methods:**

One hundred eighty three isolates of *Mycobacterium tuberculosis *from 80 HIV seropositive and 103 HIV seronegative patients were fingerprinted by standard IS*6110*-RFLP. Using the BioNumerics software, strains were considered to be clustered if at least one other patient had an isolate with identical RFLP pattern.

**Results:**

One hundred and eighteen different fingerprint patterns were obtained from the 183 isolates. There were 34 clusters containing 54% (99/183) of the patients (average cluster size of 2.9), and a majority (96.2%) of the strains possessed a high copy number (≥ 5 copies) of the IS*6110 *element. When strains with <5 bands were excluded from the analysis, 50.3% (92/183) were clustered, and there was no difference in the level of diversity of DNA fingerprints observed in the two sero-groups (adjusted odds ratio [aOR] 0.85, 95%CI 0.46–1.56, *P *= 0.615), patients aged <40 years (aOR 0.53, 95%CI 0.25–1.12, *P *= 0.100), and sex (aOR 1.12, 95%CI 0.60–2.06, *P *= 0.715).

**Conclusion:**

The sample showed evidence of a high prevalence of recent transmission with a high average cluster size, but infection with an isolate with a fingerprint found to be part of a cluster was not associated with any demographic or clinical characteristics, including HIV status.

## Background

Tuberculosis (TB) remains a major health problem in most of sub-Saharan Africa. In March 2008, the incidence and mortality associated with tuberculosis in Uganda were 355 cases and 84 deaths per 100,000 population per year, respectively [1]. In this setting, a further danger lies in the existence of TB/HIV co-infected individuals. Kampala, the capital city of Uganda, has a population of over two million people (National Housing and Census Survey, 2002) and carries 30% of the TB burden of the country (National TB and Leprosy Programme records). Records from the National referral hospital, Mulago, Kampala, show that 50% of TB patients are co-infected with HIV. Rubaga, one of the five administrative divisions surrounding the business capital of Kampala, has a resident catchment population of about 178,309 people and an average population density of 4,000/sq km (projection from the National Housing and Census Survey, 2002). About 250–300 TB cases are diagnosed from the TB clinics in the division quarterly (National TB and Leprosy Programme records). Poor housing conditions and overcrowding in most parishes of the division could facilitate the transmission of TB in the communities.

Molecular genotyping tools have enhanced our understanding of the epidemiology of TB by providing insight into the transmission dynamics, source and spread of *M. tuberculosis *[2,3]. By helping to differentiate an ongoing exogenous infection from reactivation, these tools also help in evaluating TB control and treatment programmes in a given setting [4]. One such genotyping tool, IS*6110 *Restriction Fragment Length Polymorphism (IS*6110*-RFLP) analysis, exploits variability in both the number and genomic position of the transposable element IS*6110 *to generate strain-specific patterns after *Pvu*II restriction digestion of genomic DNA [5]. Because it has been observed that the molecular clock for IS*6110*-based RFLP runs at a faster rate than that for the PCR-based Spoligotyping and Mycobacterial Interspersed Repetitive Unit (MIRU) typing techniques [6], IS*6110*-RFLP is still referred to as the gold standard molecular epidemiological tool for TB in spite of its being labour intensive. In this study, we have used standard IS*6110*-RFLP [5] to determine the rate of recently transmitted TB in Rubaga division so as to understand the dynamics of transmission of the disease in this community. The findings are related to the HIV sero-status of the patients, socio-demographic characteristics of the patients, as well as drug susceptibility pattern of the isolates.

## Methods

### Setting and study population

Rubaga, one of the five administrative divisions of Kampala city, covers an area of approximately 30 km^2^. It is located in the western part of Kampala (Latitude: 0°18' 0 N, Longitude: 32°32' 60 E). The district is also home to two missionary founded hospitals (Rubaga and Mengo) and two other public health care clinics (Namungoona health centre and JOY medical centre) that offer TB diagnostic and treatment services to patients under the supervision of the National TB and Leprosy Programme (NTLP). A sample of 344 newly presenting TB patients was enrolled at the four sites above from the resident population of Rubaga division between February and September 2006 so as to determine the species and strain diversity of *M. tuberculosis *complex in this division [7,8]. Of 344 subjects enrolled, 92 (26.7%) were HIV seropositive, 176 (51.2%) seronegative, while 76 (22.1%) did not consent to HIV testing and hence their status unknown. In the current study, we have fingerprinted 183 isolates from newly presenting patients with known HIV serostatus. Twelve isolates from HIV seropositive patients did not have adequate DNA for RFLP, so strains from only 80 HIV seropositive were analyzed, while 103 strains from HIV-seronegative individuals whose isolates had sufficient growth on sub-culture were fingerprinted so as to study the dynamics of TB transmission in HIV co-infected and non-HIV co-infected patients in this setting by analysing clustering by IS*6110 *RFLP. For the entire sample of 344 patients, 163 (47.4%) were from female patients while 181 (52.6%) were from male patients. In the current subset, 81 (44.3%) of the isolates were from female patients while 102 (55.7%) were from male patients. Five isolates were resistant to isoniazid only while another five were resistant to both isoniazid and rifampicin, hence multidrug resistant. All rifampicin resistant isolates were also resistant to isoniazid.

### Ethical considerations

Institutional permission to conduct the study was obtained from the Faculty Research and Ethics Committee of Makerere University Medical School (institutional ethics board of the Medical School). Informed consent to participate in the study as well as permission to use isolates from samples provided were obtained from all enrolled participants.

### Demographic characteristics, drug susceptibility testing and HIV sero-status

These data were available from our previous analyzes of the sample set [7,8].

### IS6110-RFLP typing and clustering analysis

Heat killed isolates were shipped to the Swedish Institute for Infectious Disease Control, Stockholm. Chromosomal DNA extraction and IS*6110*-RFLP genotyping were performed in accordance with a standard protocol described previously [5], using an IS*6110 *right-side-specific probe. BioNumerics (v 5.1) software (Applied Maths, Sint Maarten Latem, Belgium) was used to analyze molecular typing results. IS*6110*-RFLP patterns were analyzed as fingerprint types and the pair wise distance between patterns was computed using unweighted pair-group method using arithmetic averages (UPGMA) and the Jaccard index [9]. Clusters were defined as at least two *M. tuberculosis *strains with identical RFLP profiles isolated from different patients. For strains with less than five bands, previous spoligotyping data was used to analyze clustering [7].

### Statistical analysis

Statistical associations between clustering, drug susceptibility data, HIV sero-status and socio-demographic characteristics were generated by Stata 8.0 using the Pearson's chi-square test. Odds ratios were estimated at 95% confidence intervals, and a *P *value of <0.05 was considered evidence of a significant difference.

## Results

### Study population

A total of 183 isolates were fingerprinted, 81 from female patients, 34 (41.9%) of whom were HIV seropositive; and 102 from males, 46 (45%) of whom were HIV seropositive. The number of patients in the 18 – 39 years age category was 144, with 64 (44.4%) of them being HIV seropositive, while the remaining 39 patients ranged between 40 and 63 years of age with 16 (41%) of them being HIV seropositive.

### Analysis of IS*6110 *clusters

**Figure 1:**
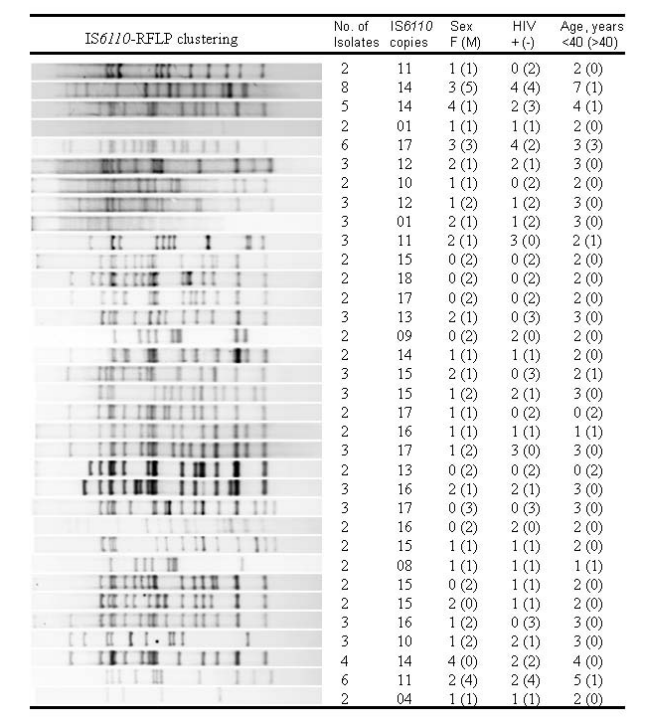
**Diversity of IS*6110*-RFLP patterns in the clustered isolates with corresponding demographic data (n = 99)**.

The RFLP pattern of the 183 isolates investigated gave 118 different IS*6110 *fingerprint patterns which included 34 genotype clusters consisting of 99 TB patients. The genotype clustering rate of the sample was 54.1% (99/183) with a median genotype cluster size of 2.5 and an interquartile range of 1. The largest genotype cluster had eight strains, followed by two clusters with six strains (Figure 1). The clustering rate among HIV seropositive patients was 52.5% (42/80) while that in the seronegative patients was 55.3% (57/103). The clustering rate within the gender was 54.3% (44/81) for females and 53.9% (55/102) for males while that within the age categories was 56.9% (82/144) for patients 18 to 39 years and 43.6% (17/39) for the 40 to 63 years category. Of the 183 samples analyzed, only five (2.7%) isolates were resistant to both rifampicin and isoniazid, defined as multi-drug resistant in this case and only one multi-drug resistant case belonged to a cluster. Bivariate analysis using odds ratio (OR) was used to compare the socio-demographic characteristics and types of patients, such as HIV sero-status, age of the patients, drug susceptibility test results of the isolates as well as spoligotypes lineages among the clustered genotypes. Having TB resistant to at least one of the two drugs tested had an OR greater than 1 but not statistically significant (*P *= 0.701), while being male, below 40 years of age and HIV seropositive each had a low OR, and were not statistically significant (Table 1). Additionally, there was no significant relationship between clustering and any of the three major groups of spoligotype lineages tested: T2 (*P *= 0.45), LAM (*P *= 0.41) and UGA (*P *= 0.47). Logistic regression analysis of the four variables showed that being male and having an isolate resistant to any of the two drugs tested had an OR greater than 1, while being HIV seropositive and below 40 years of age had an OR less than 1 (Table 2). None of clinical and patient characteristics analyzed showed a significant relationship with clustering in the sample.

**Table 1:** Association of variables with *M. tuberculosis *genotype clustering by IS*6110*-RFLP analysis (n = 183)

	Genotype clustering				
				
Demographic characteristics	No	Yes	Unadjusted OR	95%CI	*P *value
Sex					

Female	37	44	Reference		

Male	47	55	0.98	0.55–1.77	0.957, NS

Age, years					

>40	22	17	Reference		

<40	62	82	0.58	0.28–1.19	0.140, NS

HIV sero-status					

Negative	46	57	Reference		

Positive	38	42	0.89	0.49–1.60	0.702, NS

Drug susceptibility*					

Sensitive	80	93	Reference		

Any resistance	4	6	1.29	0.35–4.73	0.701, NS

OR = odds ratio; CI = confidence interval; NS = not significant; * = susceptibility to isoniazid and rifampicin only.

**Table 2:** Logistic regression analysis of the association of variables with genotype clustering by IS*6110*-RFLP for strains with >5 copies of IS*6110 *(n = 176)

Demographic characteristics	^†^**aOR**	95%CI	*P *value
Sex			

Female	Reference		

Male	1.12	0.60–2.06	0.715, NS

Age, years			

≥ 40	Reference		

≤ 40	0.53	0.25–1.12	0.100, NS

HIV sero-status			

Negative	Reference		

Positive	0.85	0.46–1.56	0.615, NS

Drug susceptibility*			

Sensitive	Reference		

Any resistance	1.44	0.38–5.38	0.586, NS

^†^Logistic regression adjusted for four variables in this table; aOR = adjusted odds ratio; CI = confidence interval; NS = not significant; * = susceptibility to isoniazid and rifampicin only.

### Analysis of IS*6110 *DNA fingerprints

**Figure 2:**
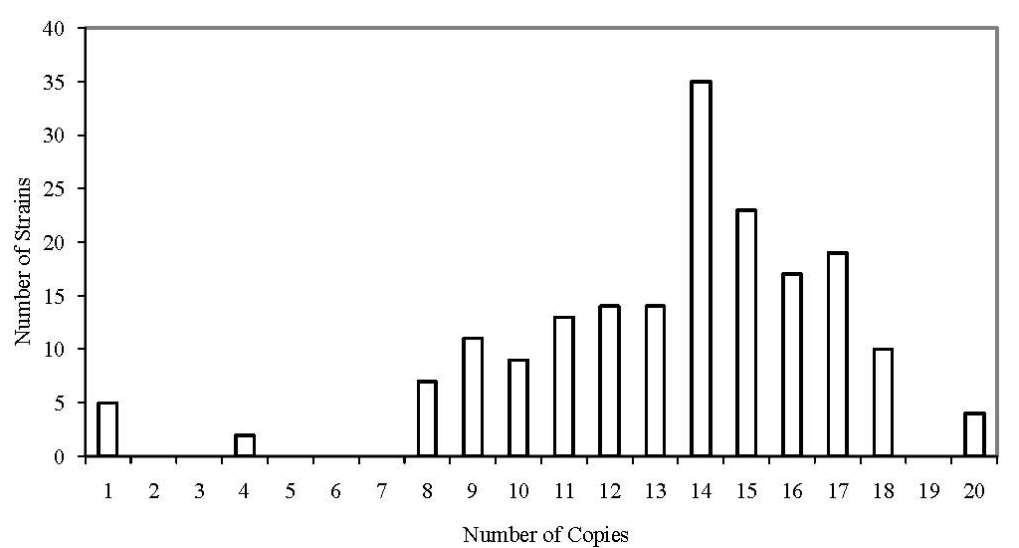
**Analysis of copy number in I*S6110*-based DNA fingerprints of *M. tuberculosis *for the 183 strains**.

The number of IS*6110 *copies for the 183 strains varied from one to 20, the mean number was 13.3 and the most frequent fingerprint pattern (n = 35) had 14 copies of IS*6110*. The proportion of strains with less than five copies (low copy number of IS*6110*) was 3.8% (7/183), 89.1% (163/183) of the strains had eight to 17 copies, while only 13 strains (7.1%) had 18 to 20 copies of IS*6110 *(Figure 2). There was no relationship between number of copies of IS*6110 *of the strains and the study variables HIV sero-status (*P *= 0.922), age (*P *= 0.063) and sex (*P *= 0.485) of the patients. Each of the seven strains that possessed less than five copies belonged to one of three clusters, 51.5% (84/163) of strains with eight to 17 copies were found in clusters, while eight of the 13 strains with more than 17 copies were clustered. The IS*6110 *copy number histogram (Figure 2) was skewed to the right with 96.2% of the strains being high copy number. Spoligotypes of the seven strains with less than five copies (data not shown) were clustered in three different patterns: two strains with four copies each were of the T2 label (spoligotype international type, SIT, 52), three strains with a single copy each were of the LAM11-ZWE label (SIT 59) while another two strains with one copy each were EAI5 (SIT 126).

### Comparison of the RFLP patterns between HIV-seropositive and HIV seronegative TB patients

The level of diversity of the DNA fingerprints observed in the HIV-seropositive TB patients was comparable to that observed in the HIV-seronegative TB group. Sixty two (77.5%) DNA fingerprint patterns were observed among the 80 isolates obtained from HIV-seropositive TB patients, and 74 patterns (71.8%) were found among 103 isolates derived from HIV-seronegative TB patients. The risk of belonging to a group of patients with identical RFLP patterns (or the risk of belonging to a chain of recent TB transmission) was 52.5% (42/80) for the HIV-seropositive group and 55.3% (57/103) for the HIV-seronegative group (*P = *0.6420). A dendrogram based on unweighted pair-group method using arithmetic averages segregated the isolates randomly with no indication of sub-structuring according to the HIV sero-status of the patients (data not shown). The number of IS*6110 *copies ranged from one to 18 for the HIV-Seropositive group and from one to 20 for the HIV-seronegative group. Analysis of the histogram (Figure 2) shows that the frequency of occurrence of IS*6110 *bands for the isolates appeared to be rather similar between the two groups being compared. For example, the most prevalent pattern observed had a peak of 14 copies of IS*6110 *(n = 35), with 17 and 18 of the strains being from HIV seropositive and HIV seronegative patients respectively.

## Discussion

The identification and differentiation of strains of *M. tuberculosis *by IS*6110*-RFLP has provided a better understanding of the epidemiology and tracing the transmission of TB in developed countries. However, not much is known in most developing countries where the disease burden is highest. Previous studies done in some sub-Saharan African countries have reported contrasting results in regard to risk factors for the transmission of TB [10-13]. In this study, we have analyzed the transmission dynamics of TB in HIV seropositive and seronegative TB patients, and within age groups so as to study the association between these variables and ability to transmit TB in a high disease burden peri-urban setting of Uganda.

Analysis of clusters in our sample showed 118 patterns from the 183 strains fingerprinted. A large proportion (99/183 or 54.1%) of our sample belonged to clusters, suggesting a high prevalence of transmission. Even after removal of clusters containing strains with less than five copies of IS*6110*, our result differs from that shown in a previous finger print analysis of 73 isolates from different villages of Kampala [14] in which it was shown that only six (8.2%) of the isolates typed clustered, suggesting that multiple strains were transmitted in the community. It is generally known that high disease burden settings are characterized by a limited variety of *M. tuberculosis *strains and a high proportion of isolates occurring in clusters [15,16]. The difference in the two results above, moreover from the same setting, could be due to the sampling strategy: while we sampled resident patients at TB clinics in a single division of Kampala, the previous study was based at the National referral hospital, with patients originating from villages of different divisions of the city. It is known that measured clustering depends on such factors as completeness of sampling, immigration and time period [17]. While our previous study sampled 2,639 suspects from a resident population of a division of Kampala, only 344 (13%) turned out smear positive and were therefore recruited [8]. Since smear microscopy has a detection limit below 50% in our setting (National TB Reference Laboratory records, Uganda), some TB cases could have been missed and therefore the true proportion of clustering in our study may have been underestimated. Additionally, in an attempt to compare an almost equal sample of HIV positive and HIV negative TB patients, only 183 of the 344 isolates (53.2%) were analyzed for clustering in the present study, a further limitation to estimating the actual clustering rate in the community studied.

Previous studies have shown varying clustering proportions dependent on length of the sampling period in settings with similar disease burdens. In South Africa for example, a one year study showed a proportion of clustering of 50% among gold miners [18]. This result is close to that obtained in the present study in which 54.1% of the isolates obtained over a 9 months period clustered. These results, however, differ from those obtained in longitudinal studies over years as they allow a greater number of exposed persons to progress to active TB. Cases in point are clustering rates of 72% in a six year study in Cape Town, South Africa, [13] and 73.6% in an area with a high HIV prevalence in Northern Malawi [11]. In a more recent analysis, it has been shown that both epidemiologic and strain related factors may contribute to cluster size [19]. It was further shown that spoligotype 59 in a Malawi population in all groups of patients, was clustered and unique, and was associated with a wide diversity of RFLP patterns. This is in agreement with our previous observation of predominance of RD 724 deleted – T2 spoligotypes of *M. tuberculosis *in this locale [8].

Our study shows that the average cluster size of 2.9 in our sample was twice that observed in another high burden setting, Tanzania, where Yang and colleagues [20] showed an average cluster size of 1.33. This difference in average cluster size between ours and the Tanzania study may be due to the fact that they sampled the city of Dar es Salaam, with patients originating from other parts of Tanzania as well, while in our study only the resident population was considered and being a non-resident of Rubaga division was an exclusion criterion. The high average cluster size in our study however, is comparable with the 3.7 observed in isolates from Addis Ababa residents in Ethiopia [10].

Only 10 of the 183 isolates were resistant to isoniazid, including five that were resistant to rifampicin as well, hence multidrug resistant. All the isolates from the sample analyzed were from newly presenting TB patients; hence the resistance of these isolates was considered primary drug resistance. There was no significant difference in the frequency of drug resistance between patients with clustered genotypes and those with unique genotypes (Table 2). Although the number of patients with strains resistant to any drug in our sample was small, this result contrasted with findings in Ethiopia in which 62.5% of 17 isolates resistant to any drug were found within clusters [10].

The relationship between clustering and sex as well as between clustering and age groups was also analyzed. Generally, as was observed in a study in Botswana [21], there was no statistical link between the risk of a patient belonging to a cluster and any of the demographic characteristics above (Table 2). These results further differ from observations in Ethiopia [10] in which there was a trend toward increased clustering of isolates from tuberculous women residing in Addis Ababa. In northern Malawi, on the other hand, there was a weak association between clustering and sex, with clustering more common in women ≥ 45 years of age [11]; while in South Africa, clustering was not associated with sex but clustered cases seemed to decrease with age, though not significantly [13].

The IS*6110 *copy number of the isolates ranged from 1 to 20, which is similar to that in the Tanzania study [20] and close to the range of 1 to 17 copies in the earlier study in Kampala [14]. These results are also comparable with that from Tunisia [22] where 75% of the isolates were high copy number. However, there were significant differences in the distribution of the copy number between our strains and those in the Tanzania sample in that the proportion of strains with less than six copies (considered low copy number strains) in this study was 3.8% (Figure 2) and 4.1% (3/73) in the previous Kampala study [14], while that in the Tanzania study was 26.1% (35/134). In fact, the isolates carrying only a single copy of the IS*6110 *element in the Tanzania study was 8%, while only 2.7% (5/183) of our strain collection and 2.7% (2/73) of the previous Kampala study harbored a single copy of the element. More interestingly, the Tanzania study found a clear correlation between the occurrence of low copy number of IS*6110 *and classification of the strains into the Asian subgroup, while in our previous analysis of the full set of 344 samples by spoligotyping, we observed that 70% (241/344) of our strain collection was of the T2 type (Euro-American) while only CAS1-Kili (3.5%) and CAS1-Delhi (2.6%) belonged to the Asian subgroup [7]. These results further confirm observations that statistically significant association of a clone to the prevalence of disease in a community may reflect increased adaptation and fitness of the strain type [23].

A majority (96.2%) of the strains in our sample and 95.9% of strains from the earlier Kampala study were high copy number, with histograms of the distribution of IS*6110 *copies from both studies skewed to the right. In northern Malawi and Botswana, on the other hand, histograms showed a normal distribution for the IS*6110 *copies. It has been observed elsewhere that high IS*6110 *copy strains can be epidemiologically resolved using a smaller set of loci on MIRU-VNTR genotyping panel [24]. Our data therefore suggests that it is possible to quickly establish epidemiological links for this group of strains using a less laborious and high throughput PCR-based technique.

A comparison of the RFLP patterns of HIV-related and non-HIV-related isolates showed no significant differences between the categories. The risk of belonging to a group of patients with identical RFLP patterns was not significantly different between the two categories (Table 2). A number of studies about clustering in TB/HIV co-infected patients have yielded contrasting results. In one of these, in a high disease incidence region of Spain, a study of 305 TB patients with known HIV serostatus showed that strains isolated from HIV-positive patients were not associated with clustering [25] a result similar to observations by Yang *et al *[20] in Tanzania. More studies in sub-Saharan Africa have pointed to absence of association between HIV serostatus and clustering. A study in a high HIV prevalence district of northern Malawi did not find any association between clustering in TB patients and HIV serostatus [19]. A similar result was obtained in a population-based prospective study of pulmonary TB patients in Botswana, in which HIV positivity was not associated with clustering even after stratification by site [21]. In Ethiopia, on the other hand, HIV positive serostatus was significantly associated with clustering of isolates for patients of both sexes [10], a result similar to that obtained in Lima, Peru, using fluorescent Amplification Fragment Length Polymorphism (fAFLP), in which the highest levels of relatedness were found among isolates from the same disease group, with the isolates grouping into two distinct clusters [23]. It may argued that observation of clonal groupings among the AIDS-associated isolates in Peru may have been due to the effect of hospitalization of the patients since all the 25 isolates from HIV-1-negative individuals were found to be relatively heterogeneous and nonclonal.

Since a majority (144/183) of the patients in our sample was aged between 18 – 39 years inclusive, the absence of association between HIV serostatus and *M. tuberculosis *clustering in this study may be due to a very high proportion of disease attributable to recent transmission in HIV negative patients in the younger category, as has been postulated elsewhere [11].

## Conclusion

The sample analyzed showed evidence of on-going transmission of TB in this setting. However, there was no statistical relationship between the risk of a patient belonging to a transmission chain and his or her HIV status, gender, age or drug susceptibility pattern of the strains. A major shortcoming of the study however, is that we included only a subset of patients diagnosed over a nine months period, and this limited the sensitivity for detecting clusters, the consequence being a low power to detect observed differences as statistically significant.

## Competing interests

The authors declare that they have no competing interests.

## Authors' contributions

BBA participated in the planning of the study, acquisition of samples and demographic data, culture and isolation of mycobacteria, RFLP assays, data analysis and drafting of manuscript; SG participated in running RFLP, data analysis and critical revision of manuscript; FAK, DPK participated in isolation of cultures and critical revision of the manuscript, TK participated in general supervision of the research in Sweden and critical revision of the manuscript; RP, AP paritcipated in running RFLP; GK & MLJ participated in the conception, design and general supervision of the study and critical revision of the manuscript. All authors read and approved the final manuscript.

## Pre-publication history

The pre-publication history for this paper can be accessed here:

http://www.biomedcentral.com/1471-2334/9/12/prepub
